# Spatiotemporal variations of public opinion on social distancing in the Netherlands: Comparison of Twitter and longitudinal survey data

**DOI:** 10.3389/fpubh.2022.856825

**Published:** 2022-07-28

**Authors:** Chao Zhang, Shihan Wang, Erik Tjong Kim Sang, Marieke A. Adriaanse, Lars Tummers, Marijn Schraagen, Ji Qi, Mehdi Dastani, Henk Aarts

**Affiliations:** ^1^Department of Psychology, Faculty of Social and Behavioral Sciences, Utrecht University, Utrecht, Netherlands; ^2^Department of Information and Computing Sciences, Faculty of Science, Utrecht University, Utrecht, Netherlands; ^3^Netherlands eScience Center, Amsterdam, Netherlands; ^4^Department of Public Governance and Management, Faculty of Social and Behavior Sciences, Utrecht University, Utrecht, Netherlands; ^5^Faculty of Science, Institute for Biodiversity and Ecosystem Dynamics, University of Amsterdam, Amsterdam, Netherlands

**Keywords:** COVID-19, social distancing, spatiotemporal analysis, public opinion, social media data, stance analysis, longitudinal survey

## Abstract

**Background:**

Social distancing has been implemented by many countries to curb the COVID-19 pandemic. Understanding public support for this policy calls for effective and efficient methods of monitoring public opinion on social distancing. Twitter analysis has been suggested as a cheaper and faster-responding alternative to traditional survey methods. The current empirical evidence is mixed in terms of the correspondence between the two methods.

**Objective:**

We aim to compare the two methods in the context of monitoring the Dutch public's opinion on social distancing. For this comparison, we quantified the temporal and spatial variations in public opinion and their sensitivities to critical events using data from both Dutch Twitter users and respondents from a longitudinal survey.

**Methods:**

A longitudinal survey on a representative Dutch sample (*n* = 1,200) was conducted between July and November 2020 to measure opinions on social distancing weekly. From the same period, near 100,000 Dutch tweets were categorized as supporting or rejecting social distancing based on a model trained with annotated data. Average stances for the 12 Dutch provinces and over the 20 weeks were computed from the two data sources and were compared through visualizations and statistical analyses.

**Results:**

Both data sources suggested strong support for social distancing, but public opinion was much more varied among tweets than survey responses. Both data sources showed an increase in public support for social distancing over time, and a strong temporal correspondence between them was found for most of the provinces. In addition, the survey but not Twitter data revealed structured differences among the 12 provinces, while the two data sources did not correspond much spatially. Finally, stances estimated from tweets were more sensitive to critical events happened during the study period.

**Conclusions:**

Our findings indicate consistencies between Twitter data analysis and survey methods in describing the overall stance on social distancing and temporal trends. The lack of spatial correspondence may imply limitations in the data collections and calls for surveys with larger regional samples. For public health management, Twitter analysis can be used to complement survey methods, especially for capturing public's reactivities to critical events amid the current pandemic.

## Introduction

The current COVID-19 pandemic caused by the SARS-CoV-2 virus has proven to be the largest challenge for public health management in recent history. Limiting contact between people through social distancing (also called “physical distancing”) was used by most countries to stop the spreading of the virus (e.g., keeping 1–2 meters distance between each other, working from home, wearing face masks at the internal public areas, etc.). In the Netherlands, the rule of keeping 1.5-meter distance from other people was in place for more than 1.5 years since the start of the pandemic. Although scientific studies have supported the effectiveness of social distancing in containing the pandemic (1), ensuring public compliance to social distancing rules is not an easy task (2, 3). After all, following social distancing measures requires people to change their natural way of interacting with others and can impede people's personal goals such as social connectedness and entertainment (4, 5). As a result, public opinion on social distancing rules is not expected to be universally positive, but to fluctuate over time, across geographical locations and social groups, and to react to policy changes and the development of the pandemic. In order to manage social distancing or other preventive measures in the current or any future epidemic, it is useful to accurately monitor public opinion on the preventive measures.

**Figure 1:**
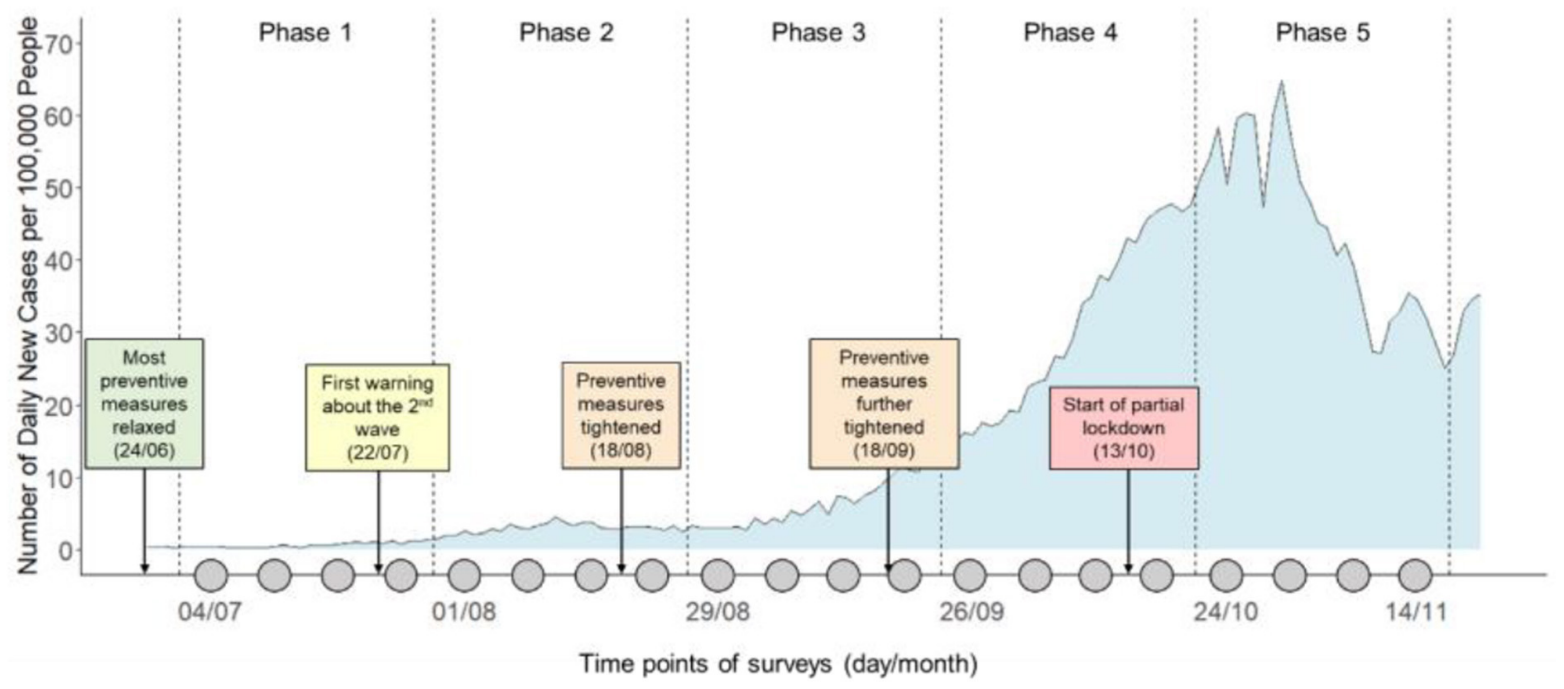
Timeline of the monitoring period in the context of the development of COVID19 pandemic in the Netherlands. Each gray dot on the timeline represents a weekly survey. The curve in the background indicates the number of daily new detected cases per 100,000 people.

In this paper, we report a study that compares Twitter analysis and a survey study in the context of monitoring public opinion on social distancing in the Netherlands. More specifically, we focused on the specific rule of *keeping 1.5 meters from other people*. Between July and November 2020, we collected tweets relating to this social distancing rule from over 96K Dutch Twitter messages and conducted a 20-week longitudinal survey about social distancing on a large (*n* = 1,200) representative Dutch sample (see Figure 1 for an illustration of the study timeline). Our objectives are 2-fold. First, we are interested in using both Twitter analysis and the longitudinal survey to gain an accurate description of the spatiotemporal variations of public opinion on social distancing in the Netherlands during the monitored period. Second, we aim to examine the similarities and differences between results obtained using the two different methods. More specifically, we ask the following questions:

1. How did Dutch people's opinions on social distancing change over time? And to what extent do Twitter and survey data agree with each other? (RQ1).
2. How did Dutch people's opinions on social distancing change spatially, i.e., across different provinces? And to what extent do Twitter and survey data agree with each other? (RQ2).
3. Are Twitter or survey data sensitive to critical events that happened in specific regions? (RQ3).

## Review of related works

### Monitoring public opinion using survey methods

The conventional way to monitor public opinion in social sciences is to run cross-sectional or longitudinal surveys, where respondents from a representative sample self-report their opinions toward an evaluative target (e.g., an election candidate or a preventive measure) (6). However, large-scale longitudinal surveys can be very expensive, and they are usually not frequent enough to detect fast changes in opinions. In addition, it is well-known that the validity of survey studies can suffer from inherent problems associated with self-reporting, such as social desirability (7, 8) and memory biases (9). In the case of social distancing, respondents may intentionally report themselves to be more supportive than they actually are, given their awareness of the social norm and the research context. Despite the limitations, conducting a survey remains the dominant method for research on public opinion relating to COVID-19 (10–13). In addition to scientific research, health organizations also use surveys to monitor public opinions in order to manage the pandemic. For example, in the Netherlands, the National Institute for Public Health and the Environment (in Dutch: Rijksinstituut voor Volksgezondheid en Milieu or RIVM) conducts such regular surveys on social distancing and other preventive measures against coronavirus.

### Monitoring public opinion using Twitter data

In recent years, mining public opinion using social media data (e.g., Twitter, Facebook, etc.) has become a popular topic in computer science and has been considered an alternative to survey methods (14–16). For example, Twitter data have been used to predict poll results for presidential election in the U.S. and consumer confidence about economy (17), showing some initial success. Natural language processing (NLP) and machine learning techniques are usually applied to automatically identify the public opinion from Twitter data (18–21). Public opinion could cover multiple perspectives. For instance, while polarity analysis considers general positive or negative attitude of tweets (14, 22), stance analysis determines the “supportive”, “against” or neutral attitude of tweets toward a target topic (e.g., governmental policies) (15, 23, 24). In other words, a person may dislike a COVID-19 measure (polarity) but still believe that people should adhere to that measure (stance). Because of this focus on beliefs and attitudes rather than only sentiments, Twitter stance analysis is especially useful for gauging whether the public supports a policy or a preventive measure in the context of COVID-19. For example, in several recent studies, researchers used Twitter stance analysis to estimate public opinion on COVID-19 vaccines (18, 25, 26). Technically, to perform a stance analysis, the classification models first need to be trained and evaluated based on manually annotated data (27, 28). Then, the validated model can be used to predict the stance of the public as an aggregate of the stance of individual messages.

Based on recent statistics, the estimated number of daily active Twitter users is 211 million worldwide and more than 1 million in the Netherlands (29). For these users, Twitter is an online platform to consume news relating to COVID-19 and to express their own opinions by posting new tweets. Given Twitter's popularity, many consider it as a cheap and accessible resource to collect large amount of data about public opinion on popular topics. While the technical aspects of storing and processing Twitter data come with some costs, the data themselves are free to a great extent – Twitter allows the free download of 1% of its public tweets (around 4.3 million) per day (30). In contrast, survey respondents usually demand financial incentives for answering questions (e.g., 0.1 euro per minute), so the cost can accumulate to be very high in a longitudinal study on a large representative sample. For example, the 20-week longitudinal study to be reported in this paper amounts to around 20,000 euro just for the respondent recruitment and survey administration alone^1^.

In addition to the considerably lower financial cost, using Twitter data can potentially transcend the two limitations of survey methods discussed earlier. First, while longitudinal surveys are usually designed to recur at a fixed interval (e.g., once a week), the data stream on Twitter is continuous (i.e., millions of new tweets every second), allowing monitoring in almost real-time (31). The much higher temporal resolution of Twitter data makes them especially suitable for detecting sudden shifts in public opinion following critical events. For example, negative opinion on social distancing following an announcement of a stricter measure may be quickly reflected in discussions and debates on Twitter. In a survey study, this shift might be observed only when the next wave of measurement is administrated or is overlooked if the shift is short-lived. Even when surveys are administrated daily, a study has shown that Twitter users responded to election campaign events 1 day earlier than survey respondents for many events (32). Second, unlike survey respondents, Twitter users are unlikely to have a homogenous motivation to respond in a socially desirable way and/or to please the researchers. In many cases, one can expect people to use Twitter as a platform to express their genuine opinion – be it positive or negative – on societal matters. Note that research has shown that Twitter users often have distinctive audience in mind (33), but they are less likely to be unequivocally biased in a socially desirable direction.

### Are Twitter data really valid for measuring public opinion?

Given the advantages discussed above, some researchers have advocated the use of Twitter analysis as a cheaper and faster alternative to surveys (17, 34–36). However, this proposition is only justifiable if stance estimated from tweets can accurately represent public opinion. There are at least two reasons to question this assumption. The first reason is inherent in Twitter as a data source itself. Although Twitter has a huge user base, its users still only form a small to moderate percentage of the population of any country, for example, <20% in the Netherlands. Even less people regularly express their opinions on COVID-19 related issues on Twitter. A bigger problem is the Twitter users' lack of representativeness of any country's population (30, 37, 38). For example, according to a 2020 survey in the Netherlands, elderly people were 2.5 times less likely to use Twitter than young adults (29). In addition to the differences in demographics, on Twitter strong opinions and extreme views are likely to be overrepresented and amplified compared to the general population (39). If a researcher's goal is to monitor public opinion that represents a country's population, there is no guarantee that Twitter data can provide such information.

The second reason for not using Twitter data relates to the underlying method of classifying tweets. Large-scale analysis relies on machine learning models that are trained to automatically detect stance based on textual data in tweets. Therefore, the validity of the method is bounded to the performance of the predictive model and the accuracy of the annotated data used to train the model. Since annotation is a subjective process, one cannot expect human annotators (usually students or crowdsourcing workers) to perfectly understand the original idea behind every tweet and disagreements are not uncommon among multiple annotators (32).

Instead of asking whether stance analysis is a valid measure of public opinion, a more sensible question might be whether it can be used to predict survey responses that are considered as the ground truth. In fact, a large portion of research on stance detection has focused on comparing their results with results from survey studies that were measured public opinion on the same subjects (30, 34, 37, 38). While earlier studies seemed to suggest strong correlations between results obtained from Twitter and survey data [e.g., consumer confidence about economy (17)], later studies failed to find universally strong correlations (40–42). A recent paper suggests that correlations between the two data sources can vary considerably depending on micro-decisions of analysis strategies (e.g., formula for calculating sentiment, different levels of smoothing for time-series) and earlier strong correlations may be spurious due to uncontrolled researcher degrees of freedom (38).

Two recent studies also compared Twitter-analytic and survey studies in the context of the COVID-19 pandemic (32, 43). Cohrdes and colleagues analyzed Twitter data from German users to monitor development of depressive symptoms during the first half of 2020 and compared the results with a 31-week national survey during the same period (43). Their results suggest strong correspondence between the two data sources for some but not all indicators of depression. Joseph and colleagues employed a more micro-level approach by modeling stance expressed in tweets from a sample of Twitter users and at the same time directly asking the same users about their opinions toward the same subjects (e.g., wearing mask, lockdown, and vaccination) (32). As the correspondence was far from perfect even for the same individuals, they concluded that stance measured using the two methods may not always reflect the same construct and different biases and measurement errors associated with the two methods contribute to the discrepancies.

In summary, the current literature suggests that Twitter analysis is more likely to complement rather than replace surveys in monitoring public opinion. From the perspective of supporting public health management, more studies are warranted to understand how the two methods can complement each other by revealing their similarities and differences, strengths and limitations. Our research serves to address this gap in the literature.

## Methods

### Collection and processing of Twitter data

The Twitter data was collected through twiqs.nl (44). It is a service provided by the Netherlands eScience Center and SURF which relies on the Twitter API to collect real-time Dutch tweets. The data collection of this project started from February 2020 (i.e. the first month a COVID-19 patient was diagnosed in The Netherlands) (45) and Dutch tweets posted between June 30 and November 16, 2020 were used in this study (i.e., consistent with the period of survey data).

**Figure 2:**
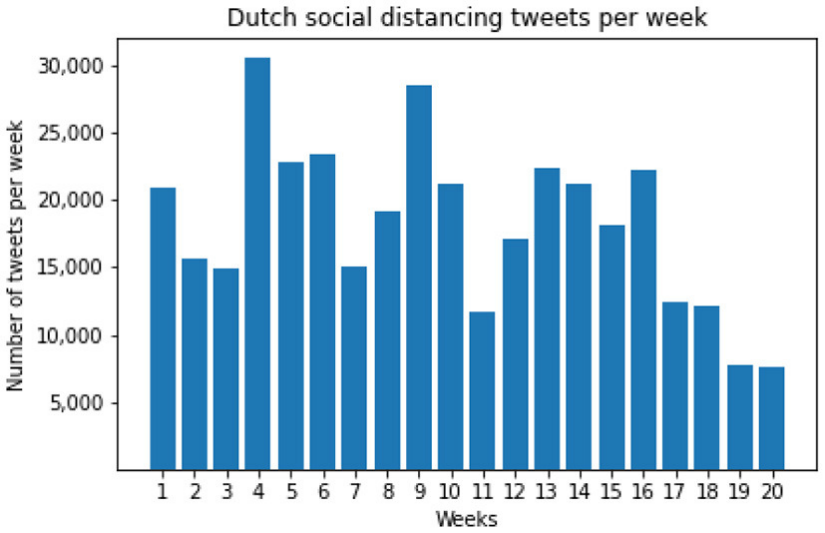
Dutch social distancing tweets per week (Week 1 is June 30 – July 6, 2020).

The Twitter corpus was first filtered to get the tweets related to the “social distancing” topic. A list of keywords was utilized to determine the related words or hashtags, including *anderhalve meter* (one and a half meter), *1.5m, 1,5m, afstand* (distance), and *hou* (keep). A preliminary processing was performed to determine these keywords for data collection [see data processing and annotation section in Wang et al. (45) for the details]. In particular, the following query (i.e., regular expression) was implemented: 1[.,] 5[-] ^*^ m | afstand. ^*^ hou | hou. ^*^ afstand | anderhalve[-] ^*^ meter (45). In total, we obtained 364,851 tweets. An overview of the tweet size in 20 weeks can be found in Figure 2.

Next, identifying the stance of every tweet in our collected data was conceptualized as a classification task. In this task, we defined three possible stances (i.e., labels) “support,” “reject,” and “other” to indicate the stance of a tweet. Here, the class “other” contains both irrelevant tweets and tweets for which the stance could not be determined (i.e., neutral). A classification model was developed based on the fastText library (46). For training this machine learning model (i.e., to classify the stance of a tweet), we conducted manual data annotation. In total, 5,977 unique tweets were randomly selected (accounting for 1% of the available data at the time of annotation) and labeled by a single annotator with respect to the question: *Does the tweet or tweet author support or reject the social distancing measure announced by the Dutch government on 15 March 2020?*. To validate the annotation results, a second annotator also annotated 400 tweets (a subset of all annotated data). The cross analysis between two annotated results reached the inter-annotator agreement κ = *0.60* (47) and shown rarely opposing annotations^2^. In total, there are 56.2% “Support” tweets, 20.0% “Reject” tweets and 23.8% “Other” tweets in the annotated data. Using the annotated data, we trained and validated the classification model using 10-fold cross-validation (80% training, 10% validation, and 10% testing). A grid search was also used to find the best values for the various fastText parameters. Next, the best trained model (with the accuracy at 65%^3^) was applied to generate the stance of every collected tweet in the 20 weeks. Here we only cover information that is essential for understanding the current research [see (27) for more details], while the trained models and the code for training and evaluating the classifier can be found online (https://github.com/puregome/models and https://github.com/puregome/notebooks/blob/master/fasttext.ipynb, respectively).

We attached locations (Dutch provinces) based on the contents of the location field in the metadata of the tweets in combination with the municipality list from Dutch Wikipedia. In particular, only the location feature in user metadata of each tweet was considered to regard their home location, for being consistent with the geographical information in the survey data. For 61.8% of the tweets, the location field was non-empty and for 30.2% of the tweets we were able to determine the province. The determination of province was done with exact match, where a place name could have different variants in the Twitter data (e.g., Den Haag, 's-Gravenhage and The Hague). Where places had the same name (e.g., Bergen), preference was given to the place with the highest number of inhabitants. In this way, we obtained a data set of 96,864 tweets, which was used in the data analysis^4^.

### Collection of longitudinal survey data

The survey data set used for the current research was collected in a larger longitudinal study on the habit formation of preventive behaviors in the context of COVID-19 pandemic. Here we only discuss details of the study that are essential for understanding the current research, but more details can be found online (https://osf.io/4utk8/). Overall, we surveyed a representative sample of the Dutch population for 20 weeks, asking them to self-report what they thought about the social distancing rule of keeping 1.5-meter distance from others. Survey respondents answered two specific questions using 9-point Likert scales: “How important do you think it is to keep 1.5 meters away from others as often as possible in the coming week?” (1 = Not at all important; 9 = Very important) and “To what extent are you motivated to keep 1.5 meters away from others as often as possible in the coming week?” (1 = Not at all motivated; 9 = Very motivated). Based on the high internal reliability (Cronbach's alpha ranged between 0.94 and 0.98 in the 20 waves), the two items were averaged to form a single measure of the respondents' attitudes toward the social distancing rule at a specific point in time. Each respondent also reported their age, gender, educational level, the place where they live, among other demographic variables. The study was approved by the Ethics Review Board of the Faculty of Social & Behavioral Sciences at Utrecht University (registration number: 20-0332).

We started the longitudinal survey with 1,200 respondents recruited through Panel Inzicht B.V. (https://panelinzicht.nl/). The sample size was determined by our goal of obtaining a large and representative Dutch sample and our budgetary constraints. A subset of the panel received email invitations to participate in the study, if they met the minimum criteria of being a Dutch national over 18 years old and living in the Netherlands. Quota sampling was used to obtain a sample that represented the Dutch population on age, gender, education level, and region of residence (see Table 1 for demographical details of the initial sample). Over the 20 weeks, the average attrition rate from week to week was around 2.1% (789 respondents for the 20th wave) and the representativeness of the sample did not change.

**Table 1:** Demographics of the initial sample.

**Variable**	**Distribution**
Age	18–34 years old: 26%; 35–54 years old: 28%; 55 years old and above: 46%
Gender	Men: 50%; women: 50%
Education level	Low: 16%; medium: 45%; high: 39%
Region of residence	Noord-Holland, Zuid-Holland, and Utrecht: 46% Groningen, Friesland, and Drenthe: 10% Overijssel, Gelderland, and Flevoland: 21% Zeeland, Noord-Brabant, and Limburg: 24%

The longitudinal study was conducted between July 4 and November 14, 2020. After the intake survey in the week before July 4, the study continued with 20 waves of online surveys on every Saturday. In each wave, an email invitation was sent to all respondents on Saturday evening at 18:00. Following the invitation, respondents had 24 h to complete the survey. Respondents used the link in the invitation email to complete the survey implemented on Qualtrics. The approximately 15-min weekly survey always started with the two questions relevant to the current paper and continued with other questions for the larger study. After completing each wave of the longitudinal survey, respondents received credits from Panel Inzicht B.V. equivalent to 70 cents as compensation. To encourage long-term engagement with the study, respondents received bonus credits equivalent to 10 euro if they managed to complete the whole study.

### Data analysis

For both the Twitter and survey data, we compute the mean and standard deviation (*SD*) of stance on social distancing for each Dutch province and each time point of monitoring. The time points were anchored on the timing of the 20 survey waves (every Saturday at 18:00). For Twitter data, all tweets with timestamps in the one-week window around the time points were used to compute the means and *SD*s for that week (from 0:00 on Tuesday to 23:50 on Monday next week)^5^. Because stance was measured with different units in the two data sources, the survey data on a 9-point scale were transformed to the interval [0, 1] to match the unit of stance estimated from tweets. After the data preprocessing, the final data frame could be understood as a 20 by 12 by 2 matrix – people's average stance (and the associated *SD*) on social distancing in 20 weeks, 12 provinces and from the two data sources.

To understand how public opinion on social distancing changes over time and across the Netherlands, we visualized the data in two ways. The first visualization focused on revealing temporal variations. For each of the 12 provinces, average stances estimated from Twitter and survey data were plotted as two 20-week time series. This visualization helped to see the temporal development of stance over time, the temporal correspondence between Twitter- and survey-based estimates, and whether these temporal patterns were consistent across the provinces. The same approach was used for visualizing the temporal change in the standard deviation of stance. The second visualization focused on revealing spatial or geographical variations. The estimates of stance were mapped onto the map of the Netherlands, with a green-red gradient indicating more supports or objections toward social distancing. In total, 40 maps were produced for the 20 weeks and the two data sources. The maps made it easier to identify potential geographical patterns in public opinion.

Data visualizations were followed by quantitative analyses. When temporal variations in stance (or its *SD*) showed linear trends, linear-mixed models were used to test the effect of time with the week indicator as the predictor and province as the grouping variable. The temporal correspondence between Twitter and survey data for each Dutch province was quantified by computing the Spearman's rank correlation coefficient. As with (43), Spearman's rank correlation was preferred to Pearson's correlation coefficient due to the small number of cases (i.e., 20 weeks). For spatial or geographical variations, linear-mixed models were first used to quantify the amount of variance that could be accounted by the different provinces for both Twitter and survey data. Next, we examined whether people's stance on social distancing was associated with the population density of the province where they lived. High population density correlates with higher infection rate (49) and may increase the relevance but also difficulty of practicing social distancing. The spatial correspondence between Twitter and survey data for each week was also quantified by Spearman's rank correlation coefficient.

Finally, we analyzed whether Twitter data and survey data were sensitive to critical events during the study period that were believed to shift public opinion on social distancing. To this end, we first selected 14 events between July and November 2020 that were related to COVID-19 from Wang et al. (45) and then asked 24 Dutch residents from Prolific (www.prolific.co) to rate the likelihood of these events to shift public opinion positively or negatively on 7-point scales (−3 = certainly negative, 0 = not sure at all, 3 = certainly positive). Based on this survey, we selected three negative events and one positive events with ratings significantly different from 0: *Dutch King and queen spend their holiday in Greece while not keeping distance* (2020-08-24; *M* = −1.83, *P* < 0.001), *Dutch Minister Grapperhaus does not keep distance at his wedding* (2020-08-27; *M* = −1.63, *P* < 0.001), *Dutch King's family travels to Greece for holiday for a second time* (2020-10-15; *M* = −1.58, *P* < 0.001), and *Donald Trump tests positive for COVID* (2020-10-02; *M* = 1.04, *P* = 0.001). Because the first two events happened in the same week, we counted them as a single event so eventually three critical timepoints were identified (week 8 to 9, 13 to 14, and 15 to 16). Welch's two-sample *t*-tests and paired *t*-tests were used for Twitter data and survey data, respectively to examine whether public opinion changed significantly in the expected direction between the pairs of two measurement points.

All the analyses were performed in the R statistical software [version 4.1.1; (50)] and with the help of the *ggplot2* (51) and *lmer* (52) packages, except for the producing of the maps. The visualization tool for generating maps was built in the JavaScript programming language and runs in a web browser. This tool was developed based on the front-end framework *React* (https://reactjs.org/tutorial/tutorial.html) and a backend data processing package *Nodejs* (https://nodejs.org). This tool creates a choropleth map of the Dutch provinces, with the values or colors of each province indicating the public's perception of the social distancing measure^6^.

## Results

### Temporal variations in public opinion and temporal correspondence between Twitter and survey data (RQ1)

Overall, stances estimated from both Twitter and survey data indicated strongly positive or supportive public opinion on social distancing between July and November 2020 in the Netherlands (grand mean: *M*_twitter_ = 0.77; *M*_survey_ = 0.84). Testing the effect of data source in a linear-mixed model suggested that stance estimated from public tweets was significantly less supportive to social distancing than stance expressed by self-report in the survey study (*B* = −0.07, 95% CI = [−0.08, −0.06], *P* < 0.001) and this difference accounted for about 22% of the variance in the dependent variable.

**Figure 3:**
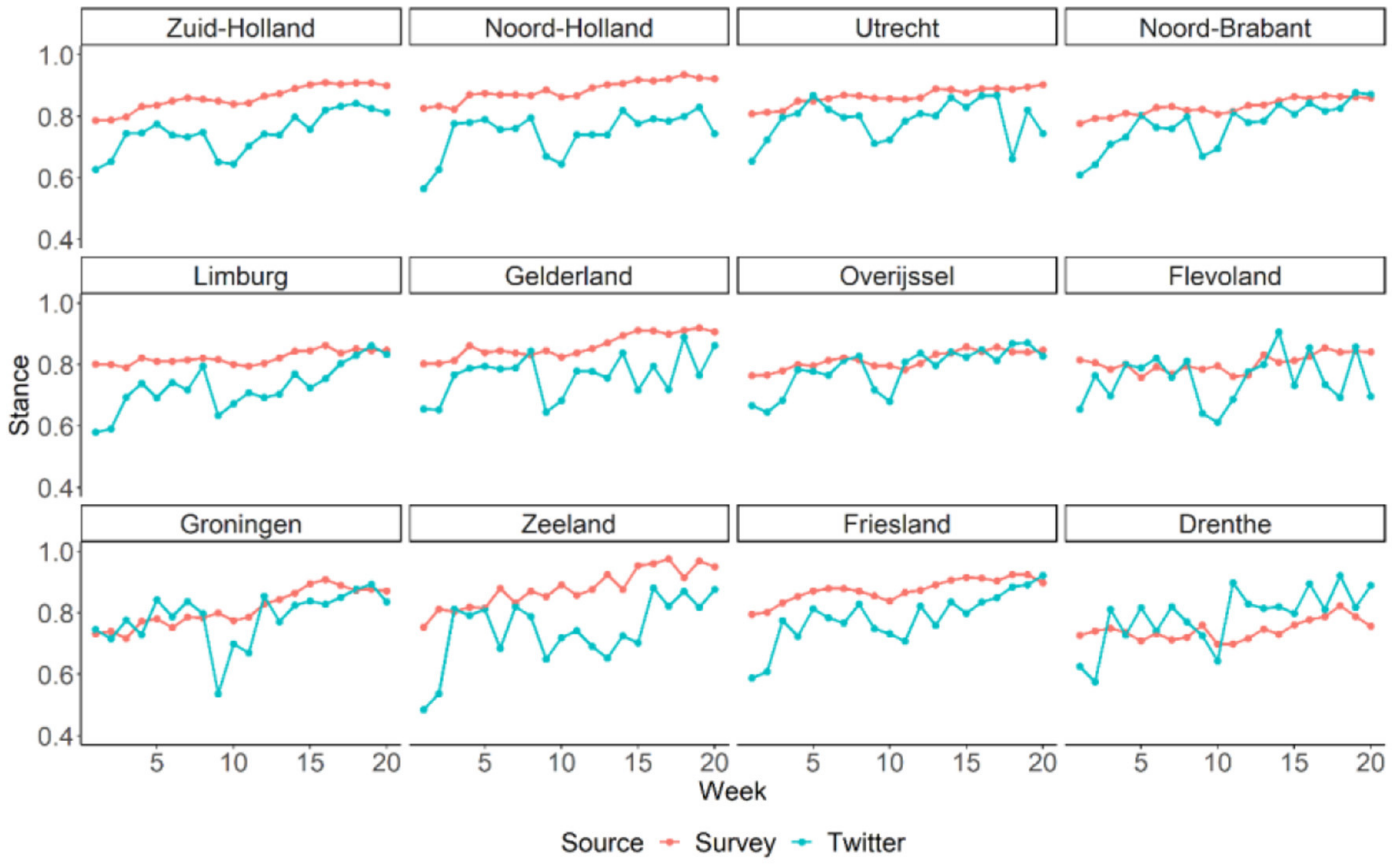
Temporal variation of stance on social distancing in Dutch provinces estimated from Twitter and survey data. Provinces are ordered from left to right and top to bottom based on their population density. The y-axis is restricted to [0.4, 1] in order to show the temporal changes more clearly.

**Figure 4:**
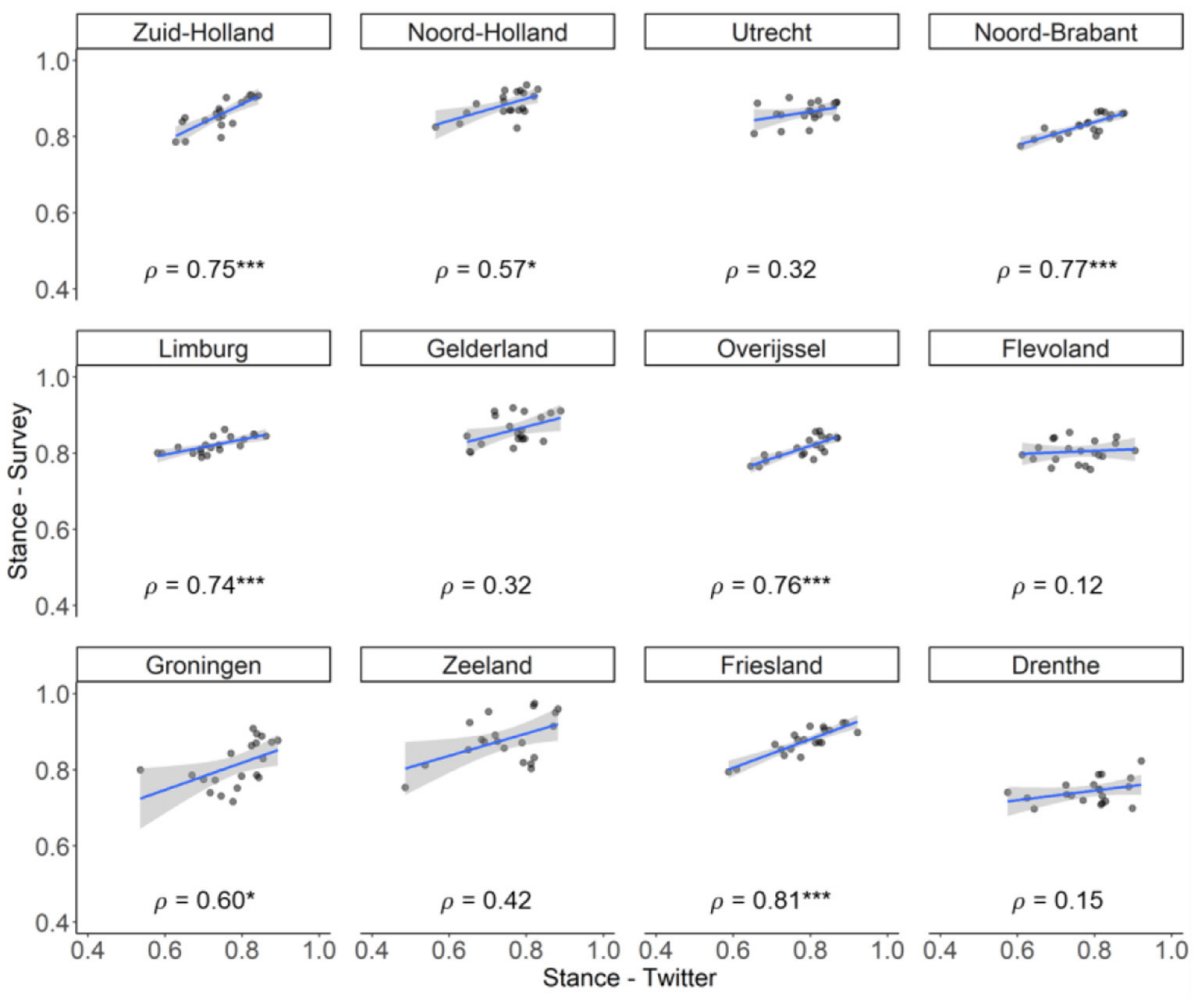
Scatterplots showing the correlations between stance estimated from Twitter and survey data for the Dutch provinces. Provinces are ordered from left to right and top to bottom based on their population density. The x- and y-axes are restricted to [0.4, 1] in order to show the temporal changes more clearly. Significance indicator: *P* < 0.05*, *P* < 0.001***.

As shown in Figure 3, there was a general trend of stance becoming more supportive over the 20 weeks, evident in both Twitter and survey data. For survey data, this increase was quite steady over time. However, for Twitter data, there were more fluctuations from week to week and there was a visible and sudden drop between the 9th and the 10th week before stance resumed to increase again. In addition, in the first few weeks, the increase in Twitter data was more dramatic, as if the time series of the Twitter data “caught up” with the time series of the survey data. The general linear increases for both data sources were confirmed by the significant positive effects of the week indicator in the linear-mixed models (for Twitter: *B* = 0.008, 95% CI = [0.006, 0.009], *P* < 0.001, Marginal *R*^2^ = 0.299; for survey: *B* = 0.005, 95% CI = [0.005, 0.006], *P* < 0.001, Marginal *R*^2^ = 0.322). Finally, as quantified by the Spearman's rank correlations (see Figure 4), stances estimated from the Twitter and survey data were significantly and strongly correlated for seven of the twelve provinces in the Netherlands.

**Figure 5:**
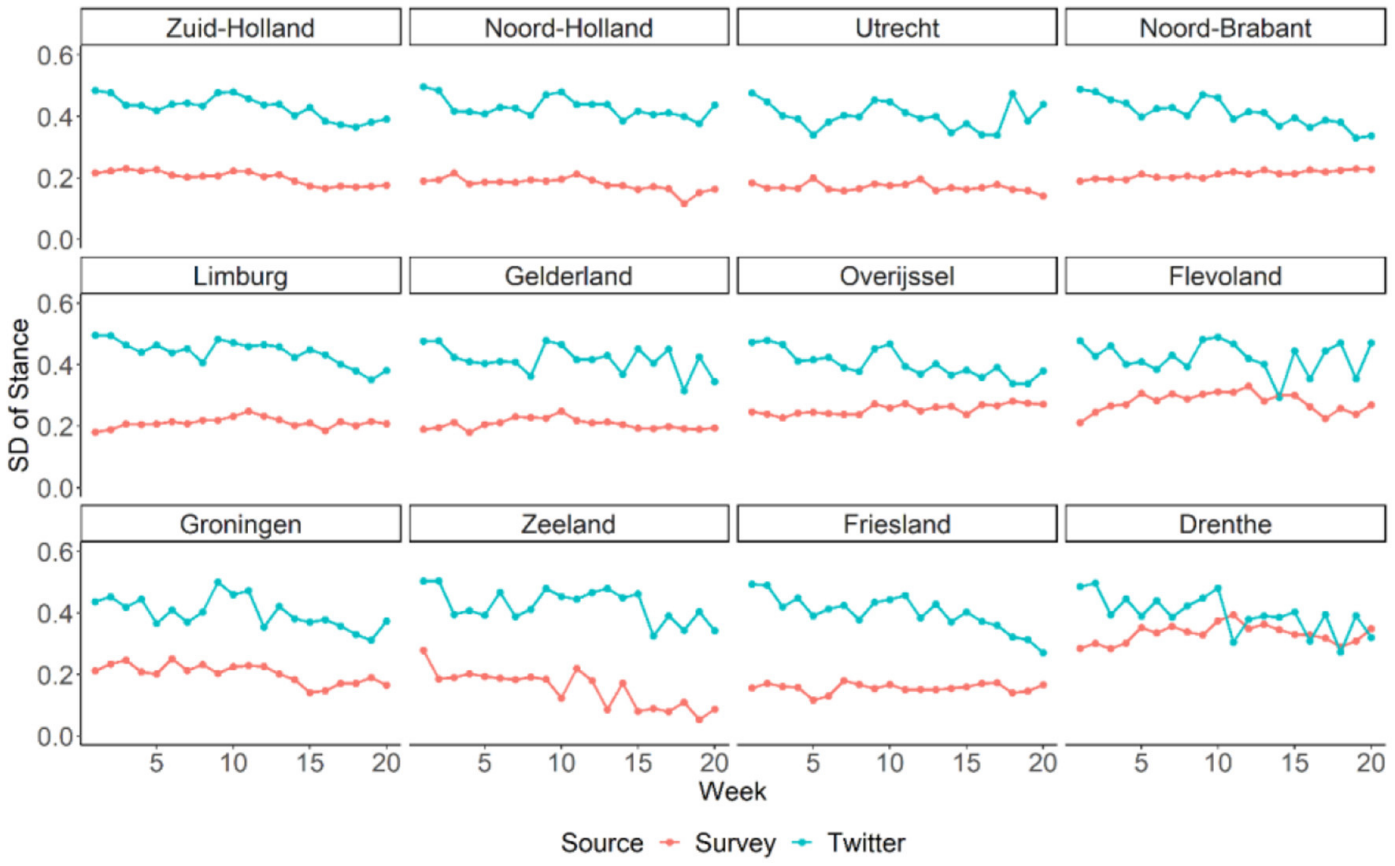
Temporal variation of standard deviation (*SD*) of stance on social distancing in Dutch provinces estimated from Twitter and survey data. Provinces are ordered from left to right and top to bottom based on their population density.

In addition to the average stance, we examined the standard deviations of stance among the tweets and the survey respondents for each week and each Dutch province (see Figure 5). Results suggested that stances varied twice as much across tweets (mean *SD* = 0.42) than across survey respondents (mean *SD* = 0.21). Moreover, the variation of stance across tweets showed gradually decrease for most of the provinces (*B* = −0.005, 95% CI = [−0.005, −0.004], *P* < 0.001, Marginal *R*^2^ = 0.302), suggesting convergence of public opinion on social distance on Twitter over time. This trend was much weaker in the survey data (*B* = −0.001, 95% CI = [−0.002, −0.001], *P* < 0.001, Marginal *R*^2^ = 0.014).

### Spatial variations in public opinion on social distancing (RQ2)

**Figure 6:**
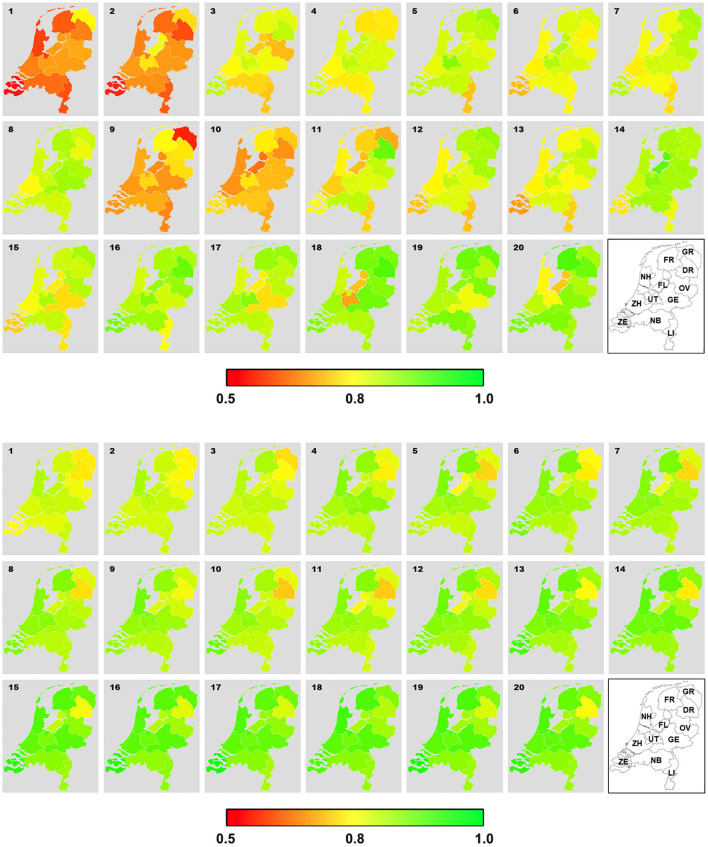
Geographical variation of stance on social distancing over the 20 weeks estimated from Twitter data (top) and survey data (bottom). ZH, Zuid-Holland; NH, Noord-Holland; UT, Utrecht; NB, Noord-Brabant; LI, Limburg; GE, Gelderland; FL, Flevoland; OV, Overijssel; GR, Groningen; ZE, Zeeland, FR, Friesland; DR, Drenthe. Redder and greener colors represent stance between 0.5 and 1.

Figure 6 basically represent the same information as Figure 3 (thus the same temporal trends can be identified by visual inspection), but they help to reveal the spatial variations of stance across the Dutch provinces. Based on Twitter data, while some differences across the provinces were visible, there were no consistent pattern over the 20 weeks. In contrast, the geographical differences in stance estimated from the survey study were rather consistent over time. For instance, it seemed clear that residents in Drenthe were almost always less supportive toward the social distancing rule than people from the rest of the country. This contrast between the two data sources in terms consistency in geographical variations was also confirmed by linear-mixed models with province as a predictor. Geographical variations could explain 52.0% of the variance in stance for survey data, but only 6.5% of the variance in stance for Twitter data. Based on the survey data, it was most notably that respondents from Drenthe (*B* = −0.12, *P* < 0.001), Flevoland (*B* = −0.06, *P* < 0.001), Groningen (*B* = −0.05, *P* < 0.001), and Overijssel (*B* = −0.05, *P* < 0.001) were less supportive for the social distancing measure than respondents from other provinces.

Furthermore, when the survey data were analyzed, results indicated that respondents who live in the provinces with higher standardized population density expressed more positive opinion on social distancing (*B* = 0.017, 95% CI = [0.012, 0.023], *P* < 0.001). However, population density only accounted for 10.3% of variance in stance, a much lower percentage than the variance accounted for when province was modeled as a categorical variable. This implied that the effect of population density was likely due to the few less populated provinces that were associated with less supportive stance (e.g., Drenthe and Groningen). When the Twitter data were analyzed, a negative correlation between standardized population density and estimated stance was found but the effect was too small to be meaningful (*B* = −0.007, 95% CI = [−0.01, −0.001], *P* = 0.035, Marginal *R*^2^ = 0.007).

Finally, results implied little spatial correspondence between Twitter and survey data in estimating public opinion on social distancing. For all 20 weeks, no significant correlation was found. The estimated Spearman's ρs fluctuated between −0.40 and 0.41, with a mean of −0.05.

### Sensitivity to critical events (RQ3)

Public opinion estimated from Twitter data shifted in the expected directions for 2 out of the 3 critical events. Corresponding to the non-compliance with the social distancing rules by the Dutch royal family and the justice minister, public opinion estimated from tweets significantly and drastically became more negative from week 8 to week 9 (mean difference = −0.134, *P* < 0.001) and seemed to recover back to the previous level after another 2 weeks. In addition, when the news of Donald Trump tested positive for coronavirus, Twitter data suggested that Dutch people did become slightly more supportive for the measure of keeping 1.5-meter distance (mean difference = 0.063, *P* = 0.003). However, while it was believed that the Dutch royal family's vacation in Greece in October should have triggered more negative opinion on social distancing, Twitter analysis did not confirm the expectation but showed a change in the opposite direction (mean difference = 0.059, *P* = 0.003).

In contrast, survey data did not confirm any of the expectations about the three critical events. First, there were no significant difference between the survey respondents' opinions in week 8 and 9 (mean difference = 0.001, *P* = 0.644). Second, survey data suggested a small decrease of attitudes toward social distancing from week 13 to 14 (mean difference = −0.01, *P* < 0.001), but this was in the opposite direction of the expectation. Finally, survey respondents' public opinion on social distancing did decrease slightly from week 15 to 16, but the difference was not statistically significant (mean difference −0.005, *P* = 0.059).

## Discussion

### Summary of findings

We monitored the spatiotemporal variations in public opinion on social distancing in the Netherlands between July and November 2020 using both stance analysis on public tweets (*n* = 364,851) and a 20-week longitudinal survey on a representative Dutch sample (initial *n* = 1,200 and final *n* = 789). When comparing the two data courses, we found that both methods indicated Dutch people to be moderately to strongly supportive of the measure of keeping 1.5-meters distance from other people during the study period and there was a largely corroborated trend of public opinion becoming more supportive over time. However, spatial or geographical variations did not show any strong pattern and the two data sources rarely mirrored each other. Results also showed that public tweets were more sensitive to critical events than survey responses.

### Twitter vs. longitudinal survey data

Following recent research on comparing Twitter and survey data (17, 30, 32, 35, 36, 38), our aim was to reveal the similarities and differences between the two data sources and to potentially understand the reasons behind them. In line with a recent study on monitoring depressive symptoms during the COVID-19 pandemic (43), our results also suggest strong temporal correspondence between public opinion monitored by Twitter analysis and survey. These matching trends in two distinct data sources provide stronger evidence that Dutch people indeed became more supportive for the measure of keeping 1.5-meters distance from July to November 2020. The correspondence is remarkable considering that the Twitter users being analyzed overlapped very little with the representative sample of longitudinal survey – in our own survey, only around 5% of the respondents used to consume corona-related contents and an even smaller 3% ever expressed their own opinions about COVID-19 on Twitter. This finding suggests that strong temporal trends in public opinion on social distancing can be tracked using both methods despite differences in samples and methodologies.

In contrast, we did not find any strong spatial correspondence between the two data sources. In addition to the underlying differences between the two methods, another contributing factor might be that there was no strong nor consistent spatial variation to begin with. The survey data did reveal significantly more negative opinion on social distancing in a few provinces but those were also the provinces with relatively small samples. Given the overall sample size, the representativeness of the sample to the Dutch population at the national level does not necessarily generalize to the regional level. If there were strong differences between the provinces (e.g., people in more populated provinces are much more in favor of or against social distancing), we would have had data to compare the two data sources on the spatial dimension more thoroughly.

Moreover, our analyses suggest that public tweets are more sensitive to news events relating to social distancing than survey responses. This contrast is consistent with a recent study where Twitter was shown to be more sensitive to critical events pertaining to several opinion targets than a survey (32). The difference in sensitivity to events may well-speak to the difference in how tweets and survey responses are generated. Twitter users spontaneously express their opinions on public matters on the platform and many tweets are likely to be direct responses to events just happened. For example, many Dutch may have directly expressed their discontent with social distancing rules after witnessing the violation by the royal family and those tweets would certainly contribute to our data and results. In surveys, however, respondents were asked to express their own opinions without referring to any events in time. Especially in our case, because the target was a behavioral measure, survey respondents possibly focused on their own motivations or intentions to keep physical distance which might be largely separable from criticisms on others.

There are two additional big differences between the Twitter and survey data. First, the average estimated public opinion from the survey study was consistently more supportive than the stance estimated from tweets. Second, estimated public opinion varied twice as much among the individual tweets than among the survey respondents. Social desirability might be one factor that contributed to the excessively supportive opinion in survey and its lack of between-respondent variability. With more diverse motivations for tweeting (33), the expressed opinions in tweets might be more varied. The difference in variability may also be accounted for by the different sampling methods for the two data sources. The longitudinal survey asked the same respondents the same set of questions every week, whereas the collected Twitter data came from a different set of users week by week.

### Implications for public health management

For the purpose of public health management, the two data sources may have their own unique uses. In our view, if the goal is to monitor the public's responses to policy changes or significant events in the context of an epidemic, analyzing public tweets is the preferred method. Understanding quick changes in stance can help to evaluate new policies and measures and to manage the aftermath of critical events. However, if the goal is to extrapolate a long-term trend in public opinion or use the monitored opinion to predict actual compliance behavior, survey data may provide higher-quality information. The accuracy of survey results does not depend on a series of error-prone procedures such as data annotation and model training. Rather, questions about people's attitudes, motivations, and intentions can be phrased precisely to obtain direct answers.

Our research also identified several strengths and limitations of both methods. Comparing to survey data, Twitter data are less susceptible to social desirability, more sensitive to critical events, and are cheaper to collect. On the other hand, our data suggest that Twitter data can suffer from limited or unbalanced availability both spatially and temporally. In the representative sample of the longitudinal study, it is by design that there were more respondents from the more populated provinces than the less populated provinces, with ratio of 11:1. This ratio is further exaggerated in terms of the number of tweets sampled from the two provinces −22,523 tweets about social distancing from Zuid-Holland but only 1,176 from Zeeland, a ratio of nearly 20:1. On the temporal dimension, as discussed in Pasek et al. (37), Twitter data may be better considered as a reflection of public attention rather than public opinion. We could collect less tweets toward the end of the study period, when Dutch people probably got used to social distancing and when wearing a face mask became the most heated topic on Twitter. If one wishes to monitor public opinion in a consistent way for a long period, longitudinal surveys may be more appropriate.

### Limitations and future work

Several limitations need to be noted when interpreting our findings. First, our ability to compare the two data sources was compromised by the different sampling methods to a certain extent. The fact that the survey respondents responded to the same questions many times might have contributed to the stability of the survey measurements. A more comparable approach to Twitter data is to survey a random sample every week. Second, despite the relatively large overall sample size for both Twitter and survey data, the sample sizes for certain less populated provinces were rather small (e.g., in the range of 20–50 tweets or survey responses per week). Thus, the estimates for public opinion in those provinces might not be accurate, which undermined our ability to reliably estimate geographical variations. Third, since only one validated stance detection model has been employed in analyzing the Twitter data, the exploration of other stance detection models [e.g., BERT (53)] is also a promising future work. Last but not least, we only monitored public opinion on one of many important preventive measures during the COVID-19 pandemic. Future research may examine whether our conclusions apply to other preventive measures in the context of the current or future epidemic.

## Data availability statement

The datasets presented in this study can be found in online repositories. The names of the repository/repositories and accession number(s) can be found below: https://osf.io/7bzhr/ and https://github.com/puregome/data.

## Ethics statement

The studies involving human participants were reviewed and approved by Ethics Review Board of the Faculty of Social and Behavioral Sciences, Utrecht University. The patients/participants provided their written informed consent to participate in this study.

## Author contributions

CZ, MA, LT, and HA were involved in the collection of the longitudinal survey data. SW, ET, MS, and MD were involved in the collection and processing of the Twitter data. JQ contributed to the data visualizations. All authors participated in writing and revising the manuscript and contributed sufficiently to be qualified as co-authors and all co-authors approved the submission.

## Funding

The Twitter data collection and analysis work was funded by Netherlands eScience Center under the project PuReGoMe (27020S04). We thank Nina Breedveld for helping with the annotation of Twitter data. The longitudinal survey study was supported by a grant awarded to HA by the Faculty of Social and Behavioral Sciences at Utrecht University for studying habits in the COVID-19 context and by the Alliance project HUMAN-AI funded by Utrecht University, Eindhoven University of Technology, Wageningen University & Research, and University Medical Center Utrecht.

## Conflict of interest

The authors declare that the research was conducted in the absence of any commercial or financial relationships that could be construed as a potential conflict of interest.

## Publisher's note

All claims expressed in this article are solely those of the authors and do not necessarily represent those of their affiliated organizations, or those of the publisher, the editors and the reviewers. Any product that may be evaluated in this article, or claim that may be made by its manufacturer, is not guaranteed or endorsed by the publisher.

## Footnotes

^1^The weekly survey in our longitudinal study took around 30 minutes to complete because we asked many more questions in addition to people's opinions on social distancing for other research purposes. Some of the questions concerning psychological processes couldn't be easily substituted by analyzing social media data.

^2^According to the standard reference for the level of agreement (48), this score at 0.6 indicates the substantial agreement of two annotators. And the majority of disagreed labels is among “other” and “support', since some tweets were supportive in an indirect way.

^3^See Wang et al. (27) and the Supplementary File for more details.

^4^The IDs of the tweets and their classified labels (including annotated ones) can be found here: https://github.com/puregome/data.

^5^The twitter data contains stance scores for each week ranging from 0 to 1. The classified stance label of each tweet is categorized (support, reject, other). Thus, we excluded the tweets with “other” label (either neural or not relevant) and computed the proportion of “support” and “reject” tweets in every week (e.g. half “reject” and half “support” = 0.5).

^6^Combined data and scripts for visualizations and statistical analyses are available at https://osf.io/7bzhr/.
