# Supplementary material for: Spatiotemporal variations of public opinion on social distancing in the Netherlands: Comparison of Twitter and longitudinal survey data
Source: Front Public Health. 2022 Jul 28;10:856825. doi: 10.3389/fpubh.2022.856825 (PMC9366395; doi:10.3389/fpubh.2022.856825)
Supplement: Supplementary file 1 [file Data_Sheet_1.PDF]

Supplementary Information

for the paper 'Spatiotemporal Variations of Public Opinion on Social Distancing in the Netherlands: Comparison of Twitter and Longitudinal Survey Data'

A. Stance classification results in the annotated Twitter data

In the section 4.2 of (25), we describe the training and testing processes of the stance classification model, as well as the parameter setting and accuracy results. Since the recall and precision results of each individual class are informative for validating our results, we present them in the below tables.

Validation data (10% of total data)

Results for individual class	Recall	Precision
Support	78.7%	73.9%
Reject	55.7%	57.5%
Other	40.8%	46.6%

Test data (10% of total data)

Results for individual class	Recall	Precision
Support	79.1%	73.5%
Reject	52.9%	58.5%
Other	41.0%	45.5%

The confusion matrix (with percentage in all data) for total data:

	predicted Other	predicted Reject	predicted Support
gold Other	9.8%	3.7%	10.3%
gold Reject	3.5%	11.1%	5.2%
gold Support	7.6%	4.4%	44.4%

From the results we can observe that the classification results regarding precision and recall tend to be consistent for each individual class. The balanced results imply that the number of false negatives is close to the number of false positives and therefore the output of our model can be considered sufficiently valid. We also observe that most of misclassification happened between 'other' vs. 'support' or 'other' vs. 'reject'. According to the confusion matrix table, among the 35% classification error, only less than 10% is caused by the confusion between support and reject. While the proportion of support vs. reject is used in the current paper in

figure 3, 4 and 6 (where the difference in our gold data and predicted data is very small), we therefore believe our stance analysis results are feasible and valid for this comparison study.
